# Novel Antifungal Dimers from the Roots of *Taiwania* *cryptomerioides*

**DOI:** 10.3390/molecules27020437

**Published:** 2022-01-10

**Authors:** Ming-Jen Cheng, Ming-Der Wu, Chao-Lin Chang, Hsun-Shuo Chang, Chiou-Fung Chyu, Yueh-Hsiung Kuo

**Affiliations:** 1 Bioresource Collection and Research Center (BCRC), Food Industry Research and Development Institute (FIRDI), Hsinchu 300, Taiwan; chengfirdi@gmail.com (M.-J.C.); wmd@firdi.org.tw (M.-D.W.); 2 Product & Process Research Center (PPRC), Food Industry Research and Development Institute (FIRDI), Hsinchu 300, Taiwan; ccl@firdi.org.tw; 3 School of Pharmacy, College of Pharmacy, Kaohsiung Medical University, Kaohsiung 807, Taiwan; hschang@kmu.edu.tw; 4 Department of Chemistry, National Taiwan University, Taipei 106, Taiwan; cmj@firdi.org.tw; 5 Department of Biotechnology, Asia University, Taichung 413, Taiwan; 6 Department of Chinese Pharmaceutical Sciences and Chinese Medicine Resources, College of Pharmacy, China Medical University, Taichung 404, Taiwan; 7 Chinese Medicine Research Center, China Medical University, Taichung 404, Taiwan

**Keywords:** *Taiwania* *cryptomerioides*, Taxodiaceae, novel skeleton, dimer, diterpenoid, antifungal activities

## Abstract

Five new dimer compounds, namely Taiwaniacryptodimers A–E (**1**–**5**), were isolated from the methanol extract of the roots of *Taiwania* *cryptomerioides*. Their structures were established by mean of spectroscopic analysis and comparison of NMR data with those of known analogues. Their antifungal activities were also evaluated. Our results indicated that metabolites **1**, **2**, **4**, and **5** displayed moderate antifungal activities against *Aspergillus* *niger*, *Penicillium* *italicum*, *Candida* *albicans*, and *Saccharomyces* *cerevisiae*.

## 1. Introduction

Taiwan cedar (*Taiwania cryptomerioides* Hayata), also known as Asia cedar, is an essential economic tree species native to Taiwan. It belongs to the same Mesozoic and Cenozoic Tertiary relics with *Ginkgo biloba*, *Sequoiadendron giganteum,* and *Metasequoia glyptostroboides*. It is a rare and precious tree species [1]. It belongs to the gymnosperm phylum, Coniferae, Coniferales, Taxodiaceae, *Taiwania*, one genus and one species. In 1904, the Japanese Prof. S. Konisi was first discovered in Nantou County, Taiwan. It was named by the botanist Prof. Hayata Bunzo in 1906 and named after Taiwan as its genus, “*Taiwania*”. The meaning of the species name “cryptomerioides” is “like Cryptomeria”. It can be seen that the morphological characteristics of Taiwan fir is similar to *Cryptomeria japonica* (L. f) D. Don. As far as the nature of the wood is concerned, Taiwan cedar is straight, delicate, and beautiful, with a light and soft texture, easy to process, and its heartwood has a beautiful yellow-red texture, which is quite distinctive. Weather resistance, decay resistance, and ant resistance are similar to those of cypress. It is a material with superior properties and is often used for construction, furniture, door panels, and thin panels. The fresh inner skin of Taiwan fir is mashed to apply it to poisonous snake bites. The leaves also have the effects of mashing juice and involving poison and are used for anti-inflammatory, diuretic, and gonorrhea treatment. 

*Taiwania cryptomerioides* (Taxodiaceae) is taxonomically included in one genus and one species of endemic plants in Taiwan. It contains more than 6% of essential oil in its heartwood [1]. *T. cryptomerioides* is a vital building material with high value in Taiwan. Earlier phytochemical investigations of this plant mainly focused on its heartwood [2,3,4] and bark constituents [5,6,7,8,9] have also been found. In the past, interesting compounds isolated from the heartwood and bark of *T. cryptomerioides* and those with biological activity prompted us to study the chemical composition of their roots. Several kinds of sesquiterpenes with unique and novel structures have also been obtained from its roots [10,11,12,13,14]. However, compared with the composition of different parts of *T. cryptomerioides* in the past literature, it is found that there is very little research on the roots, and it is worth continuing to explore its composition. In this paper, we would like to report the five new dimers, namely, Taiwaniacryptodimers A–E (**1**–**5**), isolated from the roots of the *T. cryptomerioides*. The MeOH extract of the roots of *T. cryptomerioides* was suspended in H_2_O and partitioned between H_2_O and EtOAc. The EtOAc-soluble portion was subjected to repeated silica gel column chromatography and semipreparative normal phase-HPLC to afford compounds **1**–**5**. Herein, the isolation and structure elucidation of five new compounds and their antifungal activity is described.

## 2. Results and Discussion

### 2.1. Structure Elucidation of Compounds

**Figure 1:**
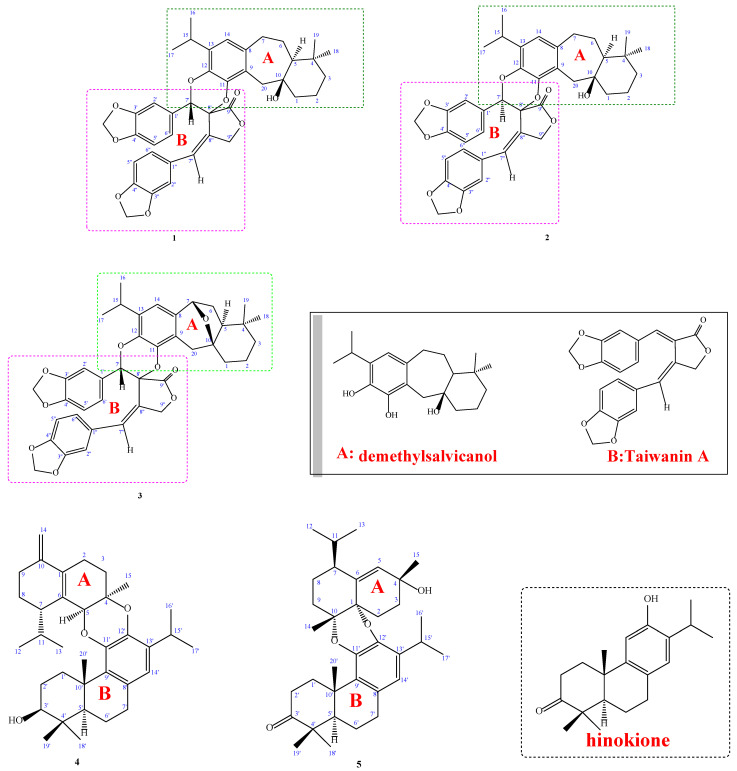
Compounds **1**–**5**, isolated from the roots of *Taiwania cryptomerioides*.

Compound **1** was isolated as gum with a specific rotation [α] ${}_{D}^{30}$ = +131.7° (*c* 0.09, CHCl_3_). The HR-EI-MS of 1 gave a molecular ion at *m*/*z* 666.2828, establishing the molecular formula of 1 as C_40_H_42_O_9_, with 20 degrees of unsaturation. The EI-MS fragmental ions of **1** at *m*/*z* 350 [C_20_H_14_O_6_]^+^ and 316 [C_20_H_2__8_O_3_]^+^, as well as forty carbon signals found in the ^13^C NMR spectrum hinted that 1 was a dimeric diterpenoid (Figure 1). The UV maxima at 229, 284, and 314 nm suggested the presence of a benzoyl functionality. The absorption bands for hydroxyl (3582 cm^−1^), γ-lactone (1791 cm^−1^), and aromatic (1616, 1499 cm^−1^) groups were observed in its IR spectrum. The ^1^H and ^13^C NMR data of 1 (Table 1 and Table 2) exhibited signals for constituent upper monomer-1 (**A**): two tertiary methyl groups [δ_H_ 0.91 and 0.88 (each 3H, s, Me-18, and Me-19], an isopropyl group attached to a phenyl group [δ_H_ 1.20 (3H, d, *J* = 6,8 Hz, Me-16), 1.24 (3H, d, *J* = 6.8 Hz, Me-17) and 3.25 (1H, sept, *J* = 6.8 Hz, H-14)], one aromatic proton [δ_H_ 6.69 (1H, s, H-11)], and a typical downshifted H_β_-1 signal of a dehydroabietane diterpene [δ_H_ 1.77 (1H, m)]; a two set contiguous structural sequence was derived from COSY correlations [δ_H_ 1.50/1.77 (each 1H, m, CH_2_-1), 1.40/1.77 (each 1H, m, CH_2_-2), 1.23/1.38 (each 1H, m, CH_2_-3); 1.30 (1H, m, H-5), 1.80/1.96 (each 1H, m, CH_2_-6)], and a nonequivalent CH_2_ was observed [δ_H_ 2.50/3.29 (each 1H, d, *J* = 14.4 Hz, CH_2_-20]. Those data proved that the structure of constituent upper monomer-1 was related to demethylsalvicanol [15]. The ^1^H and ^13^C NMR data of 1 (Table 1) also exhibited another set of signals for constituent lower monomer-2 (**B**): two methylenedioxy groups (δ_H_ 5.94 (2H, s, C4′,5′-OCH_2_O-), 5.98/6.00 (each 1H, *J* =1.6 Hz, C4″,5″-OCH_2_O-) at two benzene moieties, respectively, and CH_2_ protons in a γ-position of a γ-lactone ring (δ_H_ 3.94 (1H, dd, *J* = 13.2, 2.8 Hz, H-9″), 4.81 (1H, dd, *J* = 13.2, 2.8 Hz, H-9″). Additionally, two ABX systems (6 H) of aromatic protons were observed (δ_H_ 6.91 (1H, d, *J* = 8.0 Hz, H-2′), 6.94 (1H, s, H-6′), 6.80 (1H, d, *J* = 8.0 Hz, H-3′), and δ_H_ 6.42 (1H, s, H-6″), 6.44 (1H, d, *J* = 8.0, H-2″), 6.73 (1H, d, *J* = 8.0 Hz, H-3″)), besides two benzylic protons (δ_H_ 5.06 (1H, s, H-7′), 6.47 (1H, s, H-7″)). The ^13^C-NMR spectrum and DEPT experiment showed 20 signals including a γ-lactone carbonyl C-atom (δ_C_ 171.7 (C-9′)), twelve aromatic C-atoms, and three CH_2_ (δ_C_ 101.4 (C4′,5′-OCH_2_O-), 101.4 (C4″,5″-OCH_2_O-), 69.6 (C-9″)) and two CH groups (δ_C_ 77.4 (C-7′), 128.7 (C-7″)). The above NMR spectroscopic data of constituent lower monomer-2 are very similar to that of Taiwanin A [16], suggesting constituent lower monomer-2 was a lignanoid-derivative. Thus, the gross structure of **1** is composed of demethylsalvicanol and Taiwanin A. After calculating the degree of unsaturation, subtracting the above-inferred part, there is still one degree of unsaturation. The C-7′/C-12 and C-8′ and C-11 linkage of the two monomers in **1** was through a *O*-linkage functionality; judged by calculating the degree of unsaturation, subtracting the above inferred parts A and B, there is still one degree of unsaturation. Its molecular formula contains nine oxygen atoms and the downfield shift of H-7′ (δ_H_ 5.06) in constituent monomer-1, compared to that of the Taiwanin A analogues.

Compound **2** is also a gum with a specific rotation [α] ${}_{D}^{30}$ = −26.2 (*c* 0.08, CHCl_3_). The HR-EI-MS of **2** also showed a molecular ion at *m*/*z* 666.2823, which corresponded to the molecular formula, C_40_H_42_O_9_, indicating 20 degrees of unsaturation. The EI-MS fragmental ions of **2** at at *m*/*z* 350 [C_20_H_14_O_6_]^+^ and 316 [C_20_H_2__8_O_3_]^+^, indicated that **2** was also a dimeric diterpenoid. The IR spectrum indicated the presence of hydroxyl (3576 cm^−1^), ester carbonyl (1791 cm^−1^), and aromatic (1616, 1499, 1447 cm^−1^) groups. The UV maxima at 229, 284, and 314 nm indicated a benzoyl functionality. Comparison of ^1^H and ^13^C NMR data of **1** and **2** (Table 1 and Table 2) showed that the signals of constituent upper monomer-1 of **2** were almost the same as those of **1**; thus, the structure of constituents upper monomer-1 and lower monomer-2 were related to demethylsalvicanol and Taiwanin A, respectively.

Compounds **1** and **2** are speculated to be 9(10→20)abeoabietane type diterpenoids (demethylsalvicanol) combined with 8,8′-lignans (Taiwanin A). After calculating the degree of unsaturation, subtracting the above inferred part, there is still 1 degree of unsaturation. Therefore, the combination of the two monomer compounds should be adjacent to the oxygen atom and cyclized. The *Z*-configuration of the extra-cyclic olefinic proton at C-7″ was confirmed by the observation of the ^1^H-^1^H NOESY correlation between H-7″and H-9″ in compounds **1**–**3** (Table 3).

Compounds **1** and **2** are pure compounds obtained from different fractions, and their NMR spectra are very similar. The main difference between the two is that the optical rotation values are, respectively, positive ([α] ${}_{D}^{30}$ = +131.7) and negative ([α] ${}_{D}^{30}$ = −26.2). Although there are other chiral centers, the absolute configurations of Parts A and B have been confirmed according to the literature. Therefore, it can only be based on the large difference between the positive and negative values of the optical rotation value that the relative stereo configuration of the C-7′ position should be the opposite. Compounds **1** and **2** were a pair of C-7′ stereoisomerism. Therefore, we temporarily decided that the relative configuration at C-7′ of **1** is *β*-orientation. According to the reverse optical rotation value, the relative configuration at C-7 of **2** is proposed as α-form. However, the absolute configurations of Compounds **1** and **2** in C-7′ and C-8′ are uncertain. These data, supported by the ^13^C NMR (Table 2), DEPT, COSY (Table 3), HSQC, and HMBC (Table 3) spectra, were in agreement with a dimer system bearing two partial structures **A** and **B**. The structures of **1** and **2** were established as taiwaniacryptodimers A and B (*rel*-(3*S*,12a*S*,*Z*)-3-(benzo[d][1,3]dioxol-5-yl)-4′-(benzo[*d*][1,3]dioxol-5-ylmethylene)-12a-hydroxy-5-isopropyl-9,9-dimethyl-4′,5′,8,8a,9,10,11,12,12a,13-decahydro-2′*H*,3*H*,7*H*-spiro[benzo[5′,6′]cyclohepta[1′,2′:3,4]benzo[1,2-*b*][1,4]dioxine-2,3′-furan]-2′-one and *rel*-(3*R*,12a*S*,*Z*)-3-(benzo[d][1,3]dioxol-5-yl)-4′-(benzo[d][1,3]dioxol-5-ylmethylene)-12a-hydroxy-5-isopropyl-9,9-dimethyl-4′,5′,8,8a,9,10,11,12,12a,13-decahydro-2′*H*,3*H*,7*H*-spiro[benzo[5′,6′]cyclohepta[1′,2′:3,4]benzo[1,2-*b*][1,4]dioxine-2,3′-furan]-2′-one), respectively.

Compound **3** was obtained as an optically active colorless gum. [α] ${}_{D}^{30}$ = +105.4 (*c* = 0.6, CHCl_3_). The molecular formula was determined as C_40_H_40_O_9_ based on the [M]^+^ peak at *m*/*z* 664.2668 (calcd. 664.2673 for C_40_H_40_O_9_) in its HR-EI-MS. UV absorptions (λ_max_ bands at 227, 284, and 308 nm) confirmed the presence of a benzenoid nucleus. The IR spectrum revealed the presence of ester lactone (1791 cm^−1^), and aromatic rings (1618, 1498, and 1447 cm^−1^), respectively. Twenty-one degrees of insaturation were determined from the molecular formula, ^13^C-NMR (Table 1), and DEPT spectra. Because part of the all spectra data of **3** were similar to **1** and **2**, it is inferred from the molecular formula and mass fragments that Compound **3** is also a dimer compound. From the ^1^H-NMR spectrum, there are isopropyl groups attached to the benzene ring (δ_H_ 1.24 (d, *J* = 6.8 Hz, H-17), 1.17 (d, *J* = 6.8 Hz, H-16), 3.27 (sept, *J* = 6.8 Hz, H-15), two singlet methyl groups (δ_H_ 0.81 (s, H-18), and 0.92 (s, H-19)).

The splitting of two groups of tri-substituted benzene ring ABX patterns [δ_H_ 6.82 (1H, d, *J* = 8.0 Hz, H-3′), 6.92 (1H, d, *J* = 8.0 Hz, H-2′), 6.94 (1H, s, H-6′), and δ_H_ 6.33 (1H, s, H-6″), 6.37 (1H, dd, *J* = 8.0, 1.6 Hz, H-2″), 6.74 (1H, d, *J* = 8.0 Hz, H-3″)], two -OCH_2_O- signals [δ_H_ 5.94 (2H, s, C4′,5′-OCH_2_O-) and 5.98/6.00 (each 1H, *J* =1.6 Hz, C4″,5″-OCH_2_O-)]. In conjunction with the ^13^C-NMR spectrum, there is a lactone signal at δ_C_ 171.4 (C-9′). From the above characteristics, we know that Compound **3** is similar to **1** and **2**. The main difference is that the part A C-7 of **3** changes from a secondary carbon to an oxygenated tertiary carbon, which is a 9(10→20)abeoabietane diterpenoids combined with 8,8′-lignan. It can also be known from the IR spectrum that there is no absorption of hydroxyl groups. The C-7 and C-10 of the Part **A** form epoxide through the connection of oxygen atoms, which is also consistent with the calculated degree of unsaturation. Part A can be confirmed by comparison with the known compound brussonol (5,6-dihydroxysalviasperanol) [17,18].

The structure was further confirmed by ^13^C NMR (Table 1), DEPT, COSY (Table 3), and NOESY (Table 3) experiments. Thus, the structure of **3** was determined to be (3′S*,7′S*,8a′S*,12a′S*,*Z*)-3′-(benzo[d][1,3]dioxol-5-yl)-4-(benzo[d][1,3]dioxol-5-ylmethylene)-5′-isopropyl-9′,9′-dimethyl-4,5,7′,8′,8a′,9′,10′,11′,12′,13′-decahydro-2*H*,3′*H*-spiro[furan-3,2′-[7,12a]epoxybenzo[5′,6′]cyclohepta[1′,2′:3,4]benzo[1,2-*b*][1,4]dioxin]-2-one and was named Taiwaniacryptodimer C.

Compound **4** was isolated as a yellowish gum; its molecular formula C_35_H_50_O_3_ was established by ^13^C-NMR and HR-EI-MS data, and the eleven degrees of insaturation were determined from the molecular formula, the ^13^C-NMR spectrum, and the DEPT experiment (Table 1). Analysis of its IR spectrum suggested that **4** contains an OH group (3423 cm^−1^) and an aromatic moiety (1615, 1464 cm^−1^). The ^13^C- and ^1^H-NMR spectrum (Table 1) data together with the UV absorption bands at λ_max_ 214 and 234 nm suggested that Compound **4** is very similar to hinokione [19].

The ^1^H NMR spectrum shows that there are two isopropyl groups [δ_H_ 1.14 (d, *J* = 6.8 Hz, H-16′), 1.23 (d, *J* = 6.8 Hz, H-17′)), 3.10 (sept, *J* = 6.8 Hz, H-15′)) and δ_H_ 0.75 ((d, *J* = 6.8 Hz, H-13), 0.94 (d, *J* = 6.8 Hz, H-12), 2.28 (m, H-11)], and one pentasubstituted benzene proton at δ_H_ 6.43 (s, H-14′), one terminal double bond at δ_H_ 4.77/4.93 (each s, CH_2_-14), 4 singlet methyls at δ_H_ 0.86 (s, CH_3_-19′), 1.04 (s, CH_3_-18′), 1.28 (s, CH_3_—20′), and 1.30 (s, CH_3_-15). The ^13^C NMR spectrum shows that 10 olefinic carbons are forming a benzene ring, a terminal double bond, a four-substituted double bond, and three oxygenated carbons at δ_C_ 78.7 (C-3′), 73.5 (C-5), 71.2 (C-4). From the above signals, it is inferred that it is a dimer formed by combining two monomers (A and B). Part A is a cadinane and Part B is an abietane skeleton.

Part B can be compared with the known compound hinokione: (1) C-3′ changes from a carbonyl group to a hydroxyl group, so the ^13^C NMR spectrum also shifts from δ_C_ 220.3 to 78.7 (C-3′). (2) Except for C-2′, 3′, 4′, 11′, 12′, which are affected by different functional groups, the ^1^H NMR and ^13^C NMR data have high similarity. After calculating the degree of unsaturation, subtracting the four unsaturations in Part A and six unsaturations in Part B, there is one unsaturation left. Therefore, the combination of two monomer compounds should be an adjacent dimer to the oxygen atom and cyclized. Based on further spectral data, the structure of **4** was established to be taiwaniacryptodimer D (*rel*-(3*S*,4a*R*,9a*R*,15*S*,15b*S*,16c*S*)-8,15-diisopropyl-4,4,9a,16c-tetramethyl-12-methylene-1,2,3,4,4a,5,6,9a,10,11,12,13,14,15,15b,16c-hexadecahydronaphtho[1,2-*b*]phenanthro[3,4-*e*][1,4]dioxin-3-ol).

Compound **5** was obtained as yellowish gum; it has the formula C_35_H_50_O_4_ according to the HR-EI-MS and ^13^C-NMR data. It has an IHD of 11 as deduced from its molecular formula. The IR spectrum shows absorptions for an OH group (3453 cm^−1^), a carbonyl group (17,121 cm^−1^), and an aromatic moiety (1464 and 1421 cm^−1^). The UV spectra of **5** confirmed the presence of an aromatic group (λ_max_ 231 and 286 nm). The ^1^H NMR spectrum of **5** (Table 1) displayed resonances for two iPr moieties [δ_H_ 0.84 (d, *J* = 6.8 Hz, H-12), 0.94 (d, *J* = 6.8 Hz, H-13), 2.12 (m, H-11) and δ_H_ 1.16/1.16 (each 3H, d, *J* = 6.8 Hz, H-16′/17′), 3.14 (sept, *J* = 6.8 Hz, H-15′)], a trisubstituted double bond [δ_H_ 5.65(d(d, *J* = 2.0 Hz, H-5)], a five-substituted benzene ring [δ_H_ 6.48 (s, H-14′)], and five singlet methyl groups [δ_H_ 1.29 (s, CH_3_-15), 1.30 (s, CH_3_-14), 1.10 (s, CH_3_-19′), 1.15 (s, CH_3_-18′), 1.17 (s, CH_3_-20′)]. The ^13^C-NMR and DEPT experiments revealed that there is a carbonyl signal of δ_C_ 220.0 (C-3′), 8 olefinic carbons form a benzene ring and a trisubstituted double bond, and three oxygen-containing carbons at δ_C_ 69.5 (C-4), 75.4 (C-1), 75.8 (C-10). From the above signals, it is inferred that it is a dimer compound formed by combining two monomers. Part A is a cadinane, and B is an abietane compound. Part B can be compared with the known compound hinokione: except for C-1, 11, and 12, which are affected by different functional groups, the ^1^H NMR and ^13^C NMR spectra are very nearly the same. After calculating the degree of unsaturation, subtracting the seven unsaturations in Part B and three unsaturations in Part A, there is only one unsaturation left. Therefore, combining the two monomer compounds should be adjacent to the oxygen atom to cyclize to Compound **5**. Finally, the 2D-NMR results are shown in Table 3. Consequently, Compound **5** was named taiwaniacryptodimer E (*rel*-(4a*R*,9a*S*,12*R*,14*S*,16a*R*,17c*S*)-12-hydroxy-8,14-diisopropyl-4,4,12,16a,17c-pentamethyl-1,4,4a,5,6,11,12,14,15,16,16a,17c-dodecahydro-10*H*-naphtho[1,8a-*b*]phenanthro[3,4-*e*][1,4]dioxin-3(2*H*)-one).

### 2.2. Biological Studies

The antifungal activities of the roots of *Taiwania cryptomerioides* were tested against the following fungi: *Aspergillus niger* (BCRC-31512), *Penicillium italicum* (BCRC-30567), *Candida albicans* (BCRC-21538), and *Saccharomyces cerevisiae* (BCRC-20822). The antifungal data are shown in Table 4 and clinically used antifungal agent, ketoconazole, was used as positive control.

Our results indicated that Metabolites **1**, **2**, **4**, and **5** present moderate antifungal activities compared with ketoconazole, for which Compound **3** was weak. From the results of the antifungal tests, the following conclusions can be drawn regarding these isolates: (a) Among the novel, Dimers **1** and **2** showed antifungal activities with inhibition zones of 25, 20, 22, and 21; 20, 18, 17, and 25 mm against *Aspergillus niger* (BCRC-31512) *Penicillium italicum* (BCRC-30567), *Candida albicans* (BCRC-21538), and *Saccharomyces cerevisiae* (BCRC-20822), respectively. (b) The epoxide Dimer **3**, taiwaniacryptodimer C (**3**), exhibited weak antifungal activities against *Aspergillus niger* (BCRC-31512) and *Penicillium italicum* (BCRC-30567) strains. (c) The other type Dimers **4** and **5**, taiwaniacryptodimer D and E (**4** and **5**) indicated the effective inhibition zones of 20, 26, 19, and 20; 22, 12, 18, and 25 mm against *Aspergillus niger* (BCRC-31512) *Penicillium italicum* (BCRC-30567), *Candida albicans* (BCRC-21538), and *Saccharomyces cerevisiae* (BCRC-20822), respectively.

Compounds **1**, **2**, **4**, and **5** were further tested for their inhibitory activity against *A. niger*, *P. italicum*, *C. albicans*, and *S. cerevisiae* by using a method as described in the experimental section (Table 5). Compound **1** was found to show inhibitory activity against *A. niger* strain with MIC value 54.87 μg/mL. Compounds **2** and **4** were found to show inhibitory activity against *S. cerevisiae* or *P. italicum* strain with MIC values 58.92 and 42.78 μg/mL. Compound **5** was also revealed to show inhibitory activity against *A. niger* and *S. cerevisiae* with MIC values of 62.86 and 56.32 μg/mL, respectively. Their bioactivity was weaker than reference compound ketoconazole (with MIC values of 3.25, 6.72, 11.79, and 3.16 μg/mL for *A. niger*, *P. italicum*, *C. albicans*, and *S. cerevisiae*, respectively). No antifungal activity (MIC > 100) was observed for Compound **3** at concentrations below 100 μg/mL in this bioassay.

Among 9(10→20)abeoabietane type diterpenoids combined with 8,8′-lignans series analogues **1**–**3**, taiwaniacryptodimers C (3) (possess the C-7 and C-10 of the part A form epoxide through the connection of oxygen atoms exhibited less effective inhibition than their analogues, taiwaniacryptodimers A (1) and B (2). Compounds **1** and **2** are a pair of diastereomers, with little difference in activity. Compounds **4** and **5** are dimers that belong to the combination of abietane and cadinane skeleton compounds. It has been reported in the literature that the compounds in the heartwood of *T. cryptomerioides* have good anti-fungal and anti-wood-destroying fungus effects, which shows that the extracted components of *T. cryptomerioides* have the potential to be used as food additives or health-care medical supplies, etc. As for how to develop and utilize, further research and exploration are needed.

## 3. Materials and Methods

### 3.1. General Experimental Procedures

TLC: silica gel 60 F_254_ precoated plates (Merck, Darmstadt, German). Column chromatography (CC): silica gel 60 (70–230 or 230–400 mesh, Merck) and Spherical C18 100A Reversed Phase Silica Gel (RP-18) (particle size: 20–40 μm) (Silicycle, Quebec, Canada). HPLC: Spherical C18 column (250 × 10 mm, 5 μm) (Waters, Milford, MA, USA); LDC-Analytical-III apparatus. UV Spectra: Jasco UV-240 spectrophotometer; λ_max_ (log ε) in nm. Optical rotation: Jasco DIP-370 polarimeter; in CHCl_3_. IR Spectra: Perkin-Elmer-2000 FT-IR spectrophotometer; ν in cm^−1^. ^1^H-, ^13^C- and 2D-NMR spectra were obtained utilizing a Varian-Mercury-500 and Varian-Unity-Plus-400 instrument and reported in CDCl_3_. ^1^H and ^13^CNMR chemical shifts are reported in ppm relative to either TMS (^1^H) (δ = 0 ppm, J in Hz) as an internal standard or the residual solvent peak as following: CDCl_3_ = 7.26 (^1^H NMR), CDCl_3_ = 77.0 (^13^C NMR). ESI and HRESIMS: Bruker APEX-II mass spectrometer; in *m*/*z*.

### 3.2. Plant Material

The roots of *T. cryptomerioides* were collected from Taichung, Taiwan, in August 1996. The plant was identified by Dr. Shang-Tzen Chang, Professor of the Department of Forestry, National Taiwan University. A voucher specimen (no. 013542) has been deposited in the Herbarium of the Department of Botany of the National Taiwan University, Taipei, Taiwan.

### 3.3. Isolation and Characterization of Secondary Metabolites

Air-dried roots of *T. cryptomerioides* (15 kg) were extracted three times with MeOH (150 L) at r.t. (7 days thrice). The methanol extract was concentrated, the brown residue suspended in H_2_O (7 L) and then extracted with EtOAc, and the EtOAc fraction (365 g) subjected to CC (silica gel, hexane/EtOAc of increasing polarity (H (100)→H: E (98:2)→H: E(97:3)→H: E (95:5)→H: E (90:10)→H: E (80:20)→H: E (70:30)→H: E (60:40)→H: E (50:50)→H: E (30:70)→E (100); then using EtOAc/Acetone of increasing polarity (E (100)→E:A (70: 30)) to give 13 fractions (1–13), and each product fraction further purified by HPLC. Fraction 6 (4.4 g) was applied to a silica gel column (230–400 mesh, 40 g), eluting with a gradient of n-hexane-EtOAc, to obtain 11 fractions (6-1-6-11). Fractions 6–8 (41 mg) were applied by HPLC (10% EtOAc/CH_2_Cl_2_) to obtain Taiwaniacryptodimer E (**5**; 4.7 mg). Fraction 7 (13.2 g) was chromatographed on a silica gel column (230–400 mesh, 650 g), eluting with CH_2_Cl_2_-acetone (10:1), to give 4 fractions: Fr. 7.1~7.4. Fr. 7.1 (18.1 g) was purified by semi-preparative HPLC (10% EtOAc/CH_2_Cl_2_, flow rate 2.5 mL/min) to yield Taiwaniacryptodimers A–D (**1**–**4**; 1.0, 1.0, 7.2, and 8.5 mg).

Taiwaniacryptodimer A (**1**): gum; [α ${]}_{D}^{30}$ = +131.7 (*c* 0.09, CHCl_3_); UV (MeOH): 229.0 (4.46), 284.0 (4.19), 314.0 (4.00) nm; IR (Neat): 3582 (-OH), 1790 (ester C=O), 1615, 1498 (benzene ring) cm^−1^; ^1^H NMR (500 MHz, CDCl_3_): see Table 1; ^13^C NMR (125 MHz, CDCl_3_): see Table 2); EIMS (70 eV) *m*/*z* (%):666 ([M]^+^, 2), 368 (8), 350 (64), 84 (100); HREIMS *m*/*z* 666.2828 [M]^+^ (calcd for C_40_H_42_O_9_, 666.2830).

Taiwaniacryptodimer B (**2**): gum; [α] ${}_{D}^{30}$ = −26.2 (*c* 0.08, CHCl_3_); UV (MeOH): 229.0 (4.32), 284.0 (4.06), 314.0 (3.53) nm; IR (Neat): 3576 (-OH), 1791 (ester C=O), 1616, 1499 (benzene ring) cm^−1^; ^1^H NMR (500 MHz, CDCl_3_): see Table 1; ^13^C NMR (125 MHz, CDCl_3_): see Table 2); EIMS (70 eV) *m*/*z* (%):666 ([M]^+^, 5), 368 (8), 350 (98); HREIMS *m*/*z* 666.2823 [M]^+^ (calcd for C_40_H_42_O_9_, 666.2830).

Taiwaniacryptodimer C (**3**): gum; [α] ${}_{D}^{30}$ = +105.4 (*c* 0.6, CHCl_3_); UV (MeOH): 227 (3.34), 284 (3.11), 309 (2.98) nm; IR (Neat): 1791 (ester C=O), 1618, 1498 (benzene ring) cm^−1^; ^1^H NMR (500 MHz, CDCl_3_): see Table 1; ^13^C NMR (125 MHz, CDCl_3_): see Table 2); EIMS (70 eV) *m*/*z* (%):666 ([M]^+^, 2), 350 (95), 316 (100), 151 (76); HREIMS *m*/*z* 666.2668 [M]^+^ (calcd for C_40_H_40_O_9_, 666.2673).

Taiwaniacryptodimer D (**4**): yellow gum; [α] ${}_{D}^{30}$ = −1.3 (*c* 0.71, CHCl_3_); UV (MeOH): 214 (2.96), 234 (3.07) nm; IR (Neat): 3423 (OH), 1678 (terminal double bond), 1615, 1464 (benzene ring) cm^−1^; ^1^H NMR (500 MHz, CDCl_3_): see Table 1; ^13^C NMR (125 MHz, CDCl_3_): see Table 2); EIMS (70 eV) *m*/*z* (%): 518 ([M]^+^, 50), 202 (100), 159 (79), 73 (52); HREIMS *m*/*z* 518.3758 [M]^+^ (calcd for C_35_H_50_O_3_, 518.3762).

Taiwaniacryptodimer E (**5**): yellow gum; [α] ${}_{D}^{30}$ = +19.6 (*c* 0.39, CHCl_3_); UV (MeOH): 231 (4.10), 286 (3.42) nm; IR (Neat): 3453 (OH), 1712 (ketone), 1464, 1421 (benzene ring) cm^−1^; ^1^H NMR (500 MHz, CDCl_3_): see Table 1; ^13^C NMR (125 MHz, CDCl_3_): see Table 2); EIMS (70 eV) *m*/*z* (%): 534 ([M]^+^, 85), 516 (18), 220 (70), 205 (100), 202 (98); HREIMS *m*/*z* 534.3703 [M]^+^ (calcd for C_35_H_50_O_4_, 534.3711).

### 3.4. Antifungal Activity Assays

Test microorganisms. The in vitro antifungal activity of Compounds **1**–**5** were tested against a panel of laboratory control strains belonging to the Bioresource Collection and Research Center (BCRC), Hsinchu, Taiwan: fungal organisms *Aspergillus niger* (BCRC-31512), *Penicillium italicum* (BCRC-30567), *Candida albicans* (BCRC-21538), and *Saccharomyces cerevisiae* (BCRC-20822).

#### 3.4.1. By Disc Diffusion Assay

The antifungal susceptibility test of the isolated compounds was performed with the following strains: *Aspergillus niger*, *Penicillium italicum*, *Candida albicans*, and *Saccharomyces cerevisiae* by the disc diffusion method and applied following the CLSI M44-A, M44-S2 (for yeasts) [20,21], and M-51P (for filamentous fungi) guideline [22]. A standard disk of ketoconazole was used as a positive control, while the disk imbued with 50 μL of pure DMSO was used as a negative control. The diameters of the inhibition zones were measured in millimeters and means of a slide caliper. Each test was performed in triplicate and repeated three times, and results were analyzed for statistical significance [20,21,22].

#### 3.4.2. By Broth Dilution Assay

The MIC determination for the antifungal assay was performed according to the CLSI (Clinical and Laboratory Standard Institute) using the broth dilution assay methods [23,24,25]. Extract stock solutions and partitions were prepared in 5% DMSO, and twofold serial dilutions were prepared in RPMI in 96-well microtiter plates (Corning Incorporated, Corning, NY, USA). The final concentrations ranged from 0.98 to 2.000 g mL^−1^. Test organisms (100 μL) were added to each well in the microtiter plates. The growth control contained medium and inoculum. Blank controls contained medium only. The microtiter plates were then incubated at 35 °C and the endpoints were read after 48 h. The lowest concentration for each test compound at which color change occurred was recorded as its primary MIC value. The average of primary values from three individual tests were calculated, and that was taken as the final MIC value for each of the test compounds.

## 4. Conclusions

The research object of this research material *Taiwania cryptomerioides* (Taxodiaceae) is taxonomically included in one genus and one species of endemic plants in Taiwan. It contains more than 6% of essential oil in its heartwood. *T. cryptomerioides* is an important building material with high value in Taiwan. Previously, we investigated the chemical components of the heartwood and bark of this plant because of its antifungal and decayresistant characteristics as well as of its beautiful yellowish-red color with distinct purplish-pink streaks. The interesting compounds and those conferring biological activities isolated from the heartwood and bark of *T. cryptomerioides* prompted us to study the chemical components of its roots. In this study, we explored one novel constituent from the roots that had not been published in the past and evaluated and screened the metabolites for antifungal activity. It is found that the new dimer skeleton components **1** and **2** have moderate antifungal activity, one epoxide dimer **3** has weak activity, and two diterpenes **4** and **5** also possess moderate antifungal activities. The unique structure and antifungal activity of those have activities that make it an interesting material for further development.

## Figures and Tables

**Table 1:** ^1^H NMR data for compounds **1**–**5** in CDCl_3_ (*δ* in ppm, *J* in Hz, 500 MHz in CDCl_3_).

No	1	2	3	4	5
**Part A**					
1	1.50 (m), 1.77 (m)	1.36 (m), 1.72 (m)	1.78 (m), 1.96 (m)		
2	1.40 (m), 1.77 (m)	1.37 (m), 1.70 (m)	1.57 (m), 1.79 (m)	2.20 (m), 2.40 (m)	1.81 (m)
3	1.23 (m), 1.38 (m)	1.23 (m), 1.49 (m)	1.50 (m), 1.11 (m)	1.62 (dt, *J* = 13.2, 6.0) 2.04 (dt, *J* = 13.2, 6.0)	1.70 (m), 2.81 (m)
4					
5	1.30 (m)	1.28 (m)	1.80 (m)	4.28 (s)	5.65 (d, *J* = 2.0)
6	1.80 (m), 1.96 (m)	1.80 (m), 1.99 (m)	1.91 (m), 2.12 (m)		
7	2.69 (m), 2.79 (m)	2.70 (m), 2.77 (m)	4.89 (br. d, J = 6.4), 2.77 (m)	2.40 (m)	2.22 (br d, *J* = 13.2)
8				1.25 (m), 1.51 (m)	1.80 (m), 1.65 (m)
9				2.20 (m), 2.40 (m)	1.41 (m), 1.74 (m)
10					
11				2.28 (m)	2.12 (m)
12				0.94 (d, *J* = 6.8)	0.84 (d, *J* = 6.8)
13				0.75 (d, *J* = 6.8)	0.94 (d, *J* = 6.8)
14	6.69 (s)	6.64 (s)	6.60 (s)	4.77 (s), 4.93 (s)	1.30 (s)
15	3.25 (sept, *J* = 6.8)	3.25 (sept, *J* = 6.8)	3.27 (sept, *J* = 6.8)	1.30 (s)	1.29 (s)
16	1.20 (d, *J* = 6.8)	1.21 (d, *J* = 6.8)	1.17 (d, *J* = 6.8)		
17	1.24 (d, *J* = 6.8)	1.18 (d, *J* = 6.8)	1.24 (d, *J* = 6.8)		
18	0.88 (s)	0.88 (s)	0.81 (s)		
19	0.91 (s)	0.91 (s)	0.92 (s)		
20	2.50/3.29 (each d, *J* = 14.4)	2.50/3.29 (each d, *J* = 14.4)	2.48/2.77 (each d, *J* = 17.0)		
**Part B**					
1′				1.37 (m) 3.17 (dt, *J* = 13.6, 3.6)	2.02 (ddd, *J* = 13.2, 11.2, 2.8) 2.92 (dt, *J* = 13.2, 8.8)
2′	6.91 (d, *J =* 8.0)	6.91 (dd, *J =* 8.0, 1.6)	6.92 (d, *J =* 8.0)	1.71 (m)	2.30 (m) 2.73 (m)
3′	6.80 (d, *J =* 8.0)	6.81 (d, *J =* 8.0)	6.82 (d, *J =* 8.0)	3.27 (dd, *J* = 9.0, 6.8)	
4′					
5′				1.24 (m)	2.11 (m)
6′	6.94 (s)	6.94 (s)	6.94 (s)	1.55 (m) 1.78 (m)	1.68 (m) 1.74 (m)
7′	5.06 (s)	5.05 (s)	5.12 (m)	2.78 (m)	2.80 (m)
8′					
9′					
10′					
11′					
12′					
13′					
14′				6.43 (*s*)	6.48 (s)
15′				3.10 (sept, *J* = 6.8)	3.14 (sept, *J* = 6.8)
16′				1.14 (d, *J* = 6.8)	1.16 (d, *J* = 6.8)
17′				1.23 (d, *J* = 6.8)	1.16 (d, *J* = 6.8)
18′				1.04 (s)	1.15 (s)
19′				0.86 (s)	1.10 (s)
20′				1.28 (s)	1.17 (s)
1″					
2″	6.44 (d, *J =* 8.0)	6.42 (d, *J =* 8.0)	6.37 (dd, *J =* 8.0, 1.6)		
3″	6.73 (d, *J =* 8.0)	6.73 (d, *J =* 8.0)	6.74 (d, *J =* 8.0)		
4″					
5″					
6″	6.42 (s)	6.41 (s)	6.33 (s)		
7″	6.47 (s)	6.44 (s)	6.35 (br. s)		
8′;					
9″	3.94/4.81 (each dd, *J =* 13.2, 2.8)	3.90 (dd, *J =* 13.2, 2.8), 4.77 (dd, *J =* 13.2, 1.6)	3.90 (dd, *J =* 13.6, 2.4), 4.74 (dd, *J =* 13.6, 2.4)		
4′,5′-OCH_2_O-	5.94 (s)	5.94 (s)	5.94 (s)		
4″,5″-OCH_2_O-	5.97 (d, *J* = 1.6) 5.99 (d, *J* = 1.6)	5.98 (d, *J* = 1.6) 6.01 (d, *J* = 1.6)	5.98 (d, *J* = 1.6) 6.00 (d, *J* = 1.6)		

**Table 2:** ^13^C NMR data for compounds **1**–**5** (*δ* in ppm, 125 MHz for ^13^C NMR in CDCl_3_).

No	1	2	3	4	5
**A**					
1	42.1	42.2	30.3	133.2	75.4
2	18.8	18.7	16.0	24.3	24.6
3	42.4	42.5	31.6	29.8	34.4
4	34.3	34.4	31.8	71.2	69.5
5	58.1	58.2	50.7	73.5	133.3
6	23.8	23.8	40.0	134.9	137.7
7	36.0	36.2	75.9	42.0	42.1
8	122.1	122.1	134.7	22.3	21.5
9	137.2	138.5	118.9	30.8	32.5
10	70.9	71.2	80.1	144.6	75.8
11	141.8	140.5	140.6	21.0	26.1
12	137.3	138.0	137.6	20.1	17.0
13	134.7	134.7	134.0	17.0	22.2
14	119.0	118.2	114.6	108.4	23.0
15	26.9	27.0	26.8	22.6	16.8
16	22.7	22.8	23.1		
17	22.1	22.3	21.7		
18	21.6	21.7	26.9		
19	32.2	32.2	30.4		
20	40.6	40.5	38.8		
**Part B**					
1′	127.2	127.1	126.9	35.6	37.1
2′	107.6	108.4	107.5	28.3	34.2
3′	108.5	108.5	108.4	78.7	220.0
4′	148.0	147.9	147.7	39.3	47.2
5′	148.3	148.3	148.37	52.5	51.8
6′	121.1	121.40	121.16	19.1	20.7
7′	77.4	76.92	76.86	32.5	31.9
8′	78.6	79.08	78.78	127.5	127.6
9′	171.7	171.50	171.39	123.4	135.0
10′				39.0	37.9
11′				139.8	138.4
12′				137.4	136.9
13′				134.2	132.3
14′				118.3	118.1
15′				26.8	26.8
16′				22.2	22.2
17′				22.4	22.4
18′				28.6	28.8
19′				15.9	19.7
20′				20.1	21.8
1″	129.6	129.6	129.9		
2″	108.4	108.4	108.3		
3″	108.5	108.5	108.4		
4″	147.9	147.9	147.9		
5″	147.7	147.9	147.9		
6″	123.2	123.1	123.2		
7″	128.7	128.6	128.4		
8″	129.1	129.1	128.8		
9″	69.6	69.6	69.6		
4′,5′-OCH_2_O-	101.4	101.4	101.4		
4″,5″-OCH_2_O-	101.4	101.4	101.4		

**Table 3:** 2D-NMR data for compounds **1**–**5** in CDCl_3_ (*δ* in ppm, *J* in Hz, 500 MHz in CDCl_3_).

No	1			2			3			4			5		
**Part A**	**COSY**	**HMBC**	**NOESY**	**COSY**	**HMBC**	**NOESY**	**COSY**	**HMBC**	**NOESY**	**COSY**	**HMBC**	**NOESY**	**COSY**	**HMBC**	**NOESY**
1	2	2, 3	2 (eq)	2	2, 3	2 (eq)	2	2, 3, 10	2 (eq)						
2	1, 3	4, 10	3 (eq)	1, 3	4, 10	3 (eq)	1, 3		3 (eq)	3	3	14	3	3	15
3	2	1, 5	18	2	1, 5	18	2	1, 2, 5	18	2	1	2	2	2	15
4															
5	6			6			6				3, 6, 7, 15, 11′	3		1, 6, 7	11, 12, 13
6	7	5, 7, 8	19	7	5, 7, 8	19	7	5, 7, 8	19						
7	6		14	6		14	6		6 (eq), 14	8, 11	1	8, 11		1, 11, 12, 13	8, 12
8										7, 9	6, 10	7 (eq)		6, 9	9
9										8	10	14		1, 8	8
10															
11										7, 12, 13	6		7, 12, 13		
12										11	7		11	7	
13										11	7		11	7	
14		7, 13, 15	7, 15, 16		7, 13, 15	7, 15, 16		7, 13, 15	7, 15, 16		1, 9, 10	9 (eq)		1, 9	2
15	16, 17	12, 14		16, 17	12, 14		16, 17	12, 14			3, 5			5	2, 3
16	15		15	15		15	15		15						
17	16	13	15	16	13	15	16	13	15						
18		3, 5			3, 5			3, 5							
19		5			5			5							
20		5, 8, 9, 10, 11			5, 8, 9, 10, 11			1, 5, 8, 9, 10, 11	5						
**Part B**															
1′										2′	2′		2′	2′	20′
2′										1′		1′, 3′, 19′, 20′	1′	3′, 10′	
3′										2′	2′, 4′				
4′															
5′	6′			6′			6′			6′	3′, 7′, 10′	3′, 6′	6′	20′	6′
6′	5′	7′		5′	7′		5′	7′		5′, 7′		5′, 7′, 19′	5′, 7′	7′, 8′, 10′	
7′		2′, 8′, 8″	2′		2′, 8′, 12′, 8″	2′		2′, 8′, 12′, 8″	2′		5′, 6′, 8′, 14′	6′, 14′		6′, 9′	
8′															
9′															
10′															
11′															
12′															
13′															
14′											9′, 12′, 15′	7′, 17′		7′, 12′, 13′, 15′	17′
15′										16′, 17′	16′, 17′		16′, 17′		
16′										15′	13′		15′		
17′										15′	13′	14′	15′		
18′											3′, 5′	3′		3′, 4′, 5′, 19′	5′
19′											3′, 5′	2′, 6′, 20′		3′, 4′, 18′	6′
20′											1′, 5′, 10′	2′, 19′		5′, 9′	
1″															
2″															
3″															
4″															
5″	6″			6″			6″								
6″	5″			5″			5″								
7″		2″, 6″, 8″	9″		2″, 6″, 8″	9″		2″, 6″, 8″	9″						
8′;															
9″			2′			2′			2′						
4′,5′-OCH_2_O-															
4″,5″-OCH_2_O-															

**Table 4:** Antifungal activity of five sufficient compounds isolated from the roots of *Taiwania cryptomerioides* (diameter of the zone of growth inhibition fungicidal zone in mm, including the diameter of the disc, 8 mm).

Test Microorganism	Isolated Compounds					
	1	2	3	4	5	Ketoconazole
*A. niger*	25.2 ± 0.2	20.2 ± 1.9	12 ± 2.9	20 ± 1.8	22.5 ± 2.2	32 ± 1.2
*P. italicum*	20.0 ± 0.2	18.2 ± 1.6	11.4 ± 2.2	26.3 ± 1.1	12.3 ± 0.3	30 ± 1.4
*C. albicans*	22.2 ± 0.2	17.0 ± 0.2	–	19 ± 2.2	18.0 ± 1.3	30 ± 5.4
*S. cerevisiae*	21.2 ± 1.8	25.1 ± 1.2	–	20 ± 1.3	24.9 ± 1.4	33 ± 1.8

Inhibition zone diameter (mm); – = no Inhibition zone; Positive control (STD): ketoconazole, Each value represents the mean ± SD.

**Table 5:** MIC values of compounds **1**–**5** in μg/mL against four fungi strains.

Compounds	*A. niger*	*P. italicum*	*C. albicans*	*S. cerevisiae*
**1**	54.87 ± 6.13 ^a^	>100	>100	>100
**2**	>100	>100	>100	58.92 ± 9.30 ^a^
**3**	>100	>100	>100	>100
**4**	>100	42.78 ± 5.23 ^a^	>100	>100
**5**	62.86 ± 8.04 ^a^	>100	>100	56.32 ± 13.19 ^a^
Ketoconazole	3.25 ± 1.48 ^a^	6.72 ± 2.23 ^a^	11.79 ± 4.81 ^a^	3.16 ± 1.51 ^a^

^a^ Each value represents the mean ± SD.

## Data Availability

Not applicable.
