# Correction: Cheng et al. Novel Antifungal Dimers from the Roots of *Taiwania cryptomerioides*. *Molecules* 2022, *27*, 437

**DOI:** 10.3390/molecules27186056

**Published:** 2022-09-16

**Authors:** Ming-Jen Cheng, Ming-Der Wu, Chao-Lin Chang, Hsun-Shuo Chang, Chiou-Fung Chyu, Yueh-Hsiung Kuo

**Affiliations:** 1 Bioresource Collection and Research Center (BCRC), Food Industry Research and Development Institute (FIRDI), Hsinchu 300, Taiwan; 2 Product & Process Research Center (PPRC), Food Industry Research and Development Institute (FIRDI), Hsinchu 300, Taiwan; 3 School of Pharmacy, College of Pharmacy, Kaohsiung Medical University, Kaohsiung 807, Taiwan; 4 Department of Chemistry, National Taiwan University, Taipei 106, Taiwan; 5 Department of Biotechnology, Asia University, Taichung 413, Taiwan; 6 Department of Chinese Pharmaceutical Sciences and Chinese Medicine Resources, College of Pharmacy, China Medical University, Taichung 404, Taiwan; 7 Chinese Medicine Research Center, China Medical University, Taichung 404, Taiwan

The authors wish to make the following changes to their paper [1].

## Author List

Original:

Ming-Jen Cheng ^1,^*, Ming-Der Wu ^1^, Chao-Lin Chang ^2^, Hsun-Shuo Chang ^3^, Chiou-Fung Chyu ^4^ and Yueh-Hsiung Kuo ^4,5,6,7^

1 Bioresource Collection and Research Center (BCRC), Food Industry Research and Development Institute (FIRDI), Hsinchu 300, Taiwan; wmd@firdi.org.tw
2 Product & Process Research Center (PPRC), Food Industry Research and Development Institute (FIRDI), Hsinchu 300, Taiwan; ccl@firdi.org.tw
3 School of Pharmacy, College of Pharmacy, Kaohsiung Medical University, Kaohsiung 807, Taiwan; hschang@kmu.edu.tw
4 Department of Chemistry, National Taiwan University, Taipei 106, Taiwan; cmj@firdi.org.tw (C.-F.C.); yhkuo800@gmail.com (Y.-H.K.)
5 Department of Biotechnology, Asia University, Taichung 413, Taiwan
6 Department of Chinese Pharmaceutical Sciences and Chinese Medicine Resources, College of Pharmacy, China Medical University, Taichung 404, Taiwan
7 Chinese Medicine Research Center, China Medical University, Taichung 404, Taiwan
* Correspondence: chengfirdi@gmail.com

To be replaced with:

Ming-Jen Cheng ^1,^^†^, Ming-Der Wu ^1^, Chao-Lin Chang ^2^, Hsun-Shuo Chang ^3^, Chiou-Fung Chyu ^4,^^†^ and Yueh-Hsiung Kuo ^5,6,7,^*

1 Bioresource Collection and Research Center (BCRC), Food Industry Research and Development Institute (FIRDI), Hsinchu 300, Taiwan; chengfirdi@gmail.com (M.-J.C.); wmd@firdi.org.tw (M.-D.W.)
2 Product & Process Research Center (PPRC), Food Industry Research and Development Institute (FIRDI), Hsinchu 300, Taiwan; ccl@firdi.org.tw
3 School of Pharmacy, College of Pharmacy, Kaohsiung Medical University, Kaohsiung 807, Taiwan; hschang@kmu.edu.tw
4 Department of Chemistry, National Taiwan University, Taipei 106, Taiwan; cmj@firdi.org.tw
5 Department of Biotechnology, Asia University, Taichung 413, Taiwan
6 Department of Chinese Pharmaceutical Sciences and Chinese Medicine Resources, College of Pharmacy, China Medical University, Taichung 404, Taiwan
7 Chinese Medicine Research Center, China Medical University, Taichung 404, Taiwan
* Correspondence: kuoyh@mail.cmu.edu.tw
† These authors contributed equally to this work.

## Author Contribution

Original:

Y.-H.K. and M.-J.C. designed the research; M.-D.W., C.-F.C., H.-S.C. and M.-J.C. performed the research; C.-L.C. and Y.H.K. conducted biological assays, Y.-H.K. and M.-J.C. helped with structure elucidation, M.-J.C. organized the data and wrote the paper. All authors have read and agreed to the published version of the manuscript.

To be replaced with:

Y.-H.K. is responsible for the main research framework; Y.-H.K. and M.-J.C. designed the research; C.-F.C. performed the chemical and structure elucidation research; C.-L.C., M.-D.W. and H.-S.C. conducted antifungal assays, Y.-H.K. and M.-J.C. helped with structure elucidation, Y.-H.K. and M.-J.C. organized the data and M.-J.C. wrote the paper. All authors have read and agreed to the published version of the manuscript.

The authors apologize for any inconvenience caused and state that the scientific conclusions are unaffected. This correction was approved by the Academic Editor. The original publication has also been updated.
